# Breast Cancer Screening Based on Supervised Learning and Multi-Criteria Decision-Making

**DOI:** 10.3390/diagnostics12061326

**Published:** 2022-05-27

**Authors:** Mubarak Taiwo Mustapha, Dilber Uzun Ozsahin, Ilker Ozsahin, Berna Uzun

**Affiliations:** 1 Department of Biomedical Engineering, Near East University, Mersin 99138, Turkey; mubarak.mustapha@neu.edu.tr (M.T.M.); ilker.ozsahin@neu.edu.tr (I.O.); 2 Department of Medical Diagnostic Imaging, College of Health Science, University of Sharjah, Sharjah 27272, United Arab Emirates; 3 Operational Research Centre in Healthcare, Near East University, Nicosia 99138, Cyprus; 4 Department of Statistics, Carlos III University of Madrid, 28903 Getafe, Madrid, Spain; buzun@est-econ.uc3m.es

**Keywords:** benign, decision-making, machine learning, malignant, supervised learning

## Abstract

On average, breast cancer kills one woman per minute. However, there are more reasons for optimism than ever before. When diagnosed early, patients with breast cancer have a better chance of survival. This study aims to employ a novel approach that combines artificial intelligence and a multi-criteria decision-making method for a more robust evaluation of machine learning models. The proposed machine learning techniques comprise various supervised learning algorithms, while the multi-criteria decision-making technique implemented includes the Preference Ranking Organization Method for Enrichment Evaluations. The Support Vector Machine, having achieved a net outranking flow of 0.1022, is ranked as the most favorable model for the early detection of breast cancer. The net outranking flow is the balance between the positive and negative outranking flows. This indicates that the higher the net flow, the better the alternative. K-nearest neighbor, logistic regression, and random forest classifier ranked second, third, and fourth, with net flows of 0.0316, −0.0032, and −0.0541, respectively. The least preferred alternative is the naive Bayes classifier with a net flow of −0.0766. The results obtained in this study indicate the use of the proposed method in making a desirable decision when selecting the most appropriate machine learning model. This gives the decision-maker the option of introducing new criteria into the decision-making process.

## 1. Introduction

For many years, disease diagnosis has consistently been recognized as a significant factor for treatment through appropriate therapies. The diagnosis of a disease is still based on various physical and chemical tests. Based on the results of these tests and examinations, aspecific illness can be anticipated. Therefore, if not correctly predicted, a disease may have no chance of being cured.

Breast cancer occurs primarily in women, even though a few recorded cases in men have been recorded. Breast cancer is the most common cancer type and develops when the breast cells divide and grow uncontrollably, resulting in the aggregation of tissues called a tumor [1]. The cause of this uncontrolled growth is yet to be identified. Yet, certain risk factors such as age, gender, genetic predisposition, weight, smoking, poor diet, birth control pills, and race culminate in the occurrence of the disease [2]. Specific changes in the breast do not always indicate tumor growth, as this can also be due to pre-menstrual hormonal changes in females.

Nonetheless, if any growth persists, prompt evaluation should be performed. Breast screening is key to finding breast cancer at early and treatable stages. It involves examining the breast for evidence of breast cancer before the onset of signs or symptoms. These signs include skin changes such as redness and swelling or the presence of lumps. A breast tumor can be benign or malignant. A benign breast tumor poses no danger, while a malignant breast tumor has the potential to be dangerous to an individual’s health. Because a benign tumor grows similarly to a normal cell, it is not considered cancerous. A malignant tumor invades nearby tissues and proliferates to neighboring cells, tissues, and organs. If left unchecked, it can proliferate beyond the primary tumor and reach other body parts. 

Nearly 85% of breast cancer instances occur in women with no previous history of breast cancer. This is often due to genetic mutations and aging [3]. However, about 5–10% of breast cancer is related to an inherited gene mutation from the individual’s parents [4]. Breast cancer is the most commonly diagnosed cancer among women, with an estimated 2.3 million new cases in 2020 [5]. Breast cancer accounted for 1 in 4 cancer cases worldwide [5] and approximately 685,000 deaths globally in 2020, with most mortality in developing and low-income countries [6]. In the United States, about 325,000 breast cancer cases were recorded in 2020 [7]. As of January 2021, around 3.8 million women have an existing history of breast cancer in the United States, including cancer survivors and those recuperating [8]. In the last 30 years, great strides have been made in the early detection of breast cancer, which has significantly reduced breast cancer mortality and the quality of life of breast cancer survivors.

With its extraordinary advancement, artificial intelligence has provided new horizons for predicting, detecting, decision-making, and planning—resulting in the option for better and less costly elements of human life. Artificial intelligence can be instrumental in diagnosing diseases [9,10,11,12,13,14], increasing accuracy in diagnosis and decision-making, and even surpassing human capability. Due to limited human attention, changing mental conditions, and a wide range of human tasks and functions, the power of human diagnosis is limited in diagnosing complex and sensitive diseases. On the other hand, the growth of machine learning (ML) techniques and the increasing volume of data generated in medical diagnosis has provided the conditions for computers to play a significant role in disease diagnosis. The results of ML in recent years have solved many of the problematic issues in artificial intelligence and have created new hope in the minds of researchers.

Several supervised learning approaches for predicting breast cancer have been compared in previous studies. Asri et al. [15] conducted a study to compare the performance of support vector machine (SVM), decision tree, naive Bayes, and k-nearest neighbor (KNN) on breast cancer in Wisconsin. The study aimed to assess the correctness of classifying data based on the efficiency and effectiveness of each algorithm in terms of accuracy, precision, sensitivity, and specificity. In the study, the 10-fold cross-validation resampling technique was used. This ensured that computational time and bias were reduced while every data point was tested precisely once.

Consequently, the dataset was fitted to the model, and the effectiveness of the model was first evaluated based on time to build a model, correctly classified instances, incorrectly classified instances, and accuracy. Although the SVM model took the longest to build, it recorded the highest number of correctly classified instances with an accuracy of 97.13% and the lowest value of incorrectly classified instances. Furthermore, the models were evaluated using the kappa statistic (KS), mean absolute error (MAE), root mean squared error (RMSE), relative absolute error (RAE), and root relative squared error (RRSE). It was concluded that the SVM model proved its efficiency in breast cancer prediction and diagnosis and achieved the best performance in terms of precision and low error rate. In another study, Bayrak et al. [16] compared SVM and the artificial neural network (ANN) to predict breast cancer in its early stages using the Wisconsin breast cancer (original) dataset. Both 10-fold cross-validation and a random resampling technique were used. The random resampling technique split the dataset into 66% for training and 33% for test samples. Moreover, the SMO (sequential minimal optimization) algorithm and LibSVM were used to classify SVM, while multi-layer perceptron and voted perceptron was used as artificial neural network (ANN) classifiers in Weka software. Finally, the models were evaluated based on accuracy, precision, recall, and ROC area, and the SMO (sequential minimal optimization algorithm) model outperformed the ANN model with an accuracy of 96.9957%. Gbenga et al. [17] evaluated eight ML models to predict breast cancer using the WEKA data mining and ML simulation environment. Then, the models were evaluated using the kappa statistic (KS), mean absolute error (MAE), root mean squared error (RMSE), relative absolute error (RAE), and root relative squared error (RRSP). SVM outperformed all others with a classification accuracy of 97.07% and the lowest error rate compared to the radial based function of 96.49%, simple linear logistic regression model of 96.78%, naive Bayes of 96.48%, KNN of 96.34%, Adaboost of 96.19%, fuzzy unordered role induction algorithm of 96.78%, and decision tree J48 of 96.48%. While previous research has shown that SVM is the most accurate ML model, other comparative studies have shown that other methods are equally valid and accurate. 

Furthermore, Amrane et al. [18] compared two separate classifiers for breast cancer classification: naive Bayes and KNN. Both cross-validation and random resampling techniques were used to overcome overfitting. The models were then evaluated using accuracy, training, and test processes. The result revealed that KNN outperformed naive Bayes with an accuracy of 97.51%. These findings suggest that comparative research on these approaches is possible, provided a common dataset is used. Naji et al. [19] conducted a study using five ML algorithms to predict and diagnose breast cancer. The dataset employed for this investigation was the Wisconsin breast cancer (original) dataset. Afterward, data cleaning, attribute selection, target role setting, and features extraction followed. The dataset was split into a training (75%) and a test set (25%). Subsequently, the algorithm was fitted with the dataset and evaluated using confusion matrix, accuracy, precision, sensitivity, F1-score, and AUC. The support vector machine demonstrated its efficiency by achieving higher efficiency of 97.2%, precision of 97.5%, AUC of 96.6%, and outperforming all other algorithms.

In another study, Zebari et al. [20] propose a new method for classifying benign or malignant breast cancer from mammogram images. The region of interest (ROI) is determined using a combination of thresholding and ML. The resulting ROI was divided into five distinct blocks. The wavelet transform was used to suppress noise from each produced block based on BayesShrink soft thresholding by capturing high and low frequencies within particular sub-bands. Multiple features are extracted from each denoised block using an upgraded fractal dimension (FD) approach dubbed multi-FD (M-FD). A genetic algorithm was used to minimize the amount of extracted features. Five classifiers were trained and combined with an artificial neural network (ANN) to classify the extracted features from each block. Finally, the fusion process combined the five blocks’ outcomes to arrive at the ultimate choice. The proposed method was validated and tested using four benchmark mammography image datasets (MIAS, DDSM, breast, and BCDR). The experiment results showed that the proposed method yielded better results on the breast dataset in the single-dataset evaluation. In comparison, better results were obtained on the remaining datasets in the double-dataset evaluation. Zebari et al. [21] conducted a systematic review of computing approaches for breast cancer detection based on computer-aided diagnosis using mammogram images. The study was based on 118 publications published between 2018 and 2021 and retrieved from major scientific databases using a rigorous systematic review methodology. A general description and analysis of existing computer-aided design (CAD) systems that use ML methods and their current state based on mammogram image modalities and classification methods were found. This systematic review discusses all aspects of CAD, including pre-processing, segmentation, feature extraction, feature selection, and classification. Gaps in the literature were identified, and recommendations were made for additional research. The study concludes that a systematic review may be beneficial for doctors who employ CAD systems for breast cancer early detection, as well as researchers who wish to identify knowledge gaps and contribute to breast cancer diagnostics.

All outlined research studies and many more have consistently based model evaluation on performance evaluation metrics. None have implemented additional important metrics and performance evaluation metrics to create robust, flexible, and encompassing metrics. This raises several questions, such as, what if a decision-maker needs more crucial factors not covered by the performance evaluation metrics? What if the decision-makers are interested in the useability of the accurate model? What if concerns such as the deployability of the model are paramount to the decision-maker? All of these questions remain unanswered. Hence, a research gap has been created.

Our proposed study presents a novel approach that combines ML and multi-criteria decision-making methods. This is a first-of-its-kind approach. It enables the evaluation of machine learning models using criteria other than the typically used performance metrics in the early detection of breast cancer. After developing a classification model, it is necessary to assess the model’s predictive ability using performance metrics such as accuracy, recall, precision, and F1 score. These metrics address false positive/false negative notions, accuracy, recall, precision, and F1-score. Still, none addresses other important factors such as the model’s usability, applicability, and the impact of different factors on the model. This can be simplified to whether an “accurate model” can handle irrelevant attributes or whether a “precise model” can be used for a large dataset. When selecting a model, these are necessary factors that matter to decision-makers. These may include the number of training samples needed, the impact of feature scaling, the impact of hyper-parameter tuning, and tolerance to irrelevant attributes. As a result, MCDM methods are crucial. MCDM methods are among the most important ways to select the most suitable decision among several alternatives [22,23,24,25]. It is an effective method with a wide range of potentiality [22] across the field of artificial intelligence. Our study proposes combining and evaluating a model’s predictive, adaptability, and usability metrics using MCDM. This will provide a robust approach for decision-makers when finding the appropriate model for selection problems and ensure that decision-making tools are in the hands of decision-makers.

## 2. Materials and Methods

### 2.1. Dataset

**Figure 1:**
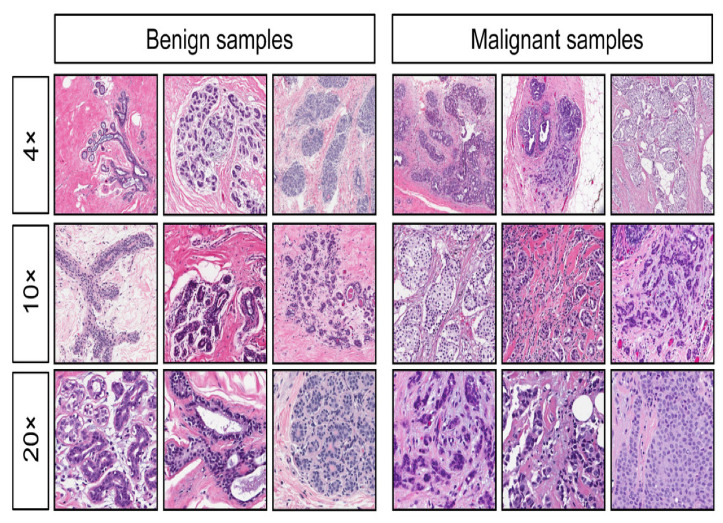
Digitalized image of the Wisconsin breast dataset [27].

We used the Wisconsin breast cancer (diagnostic) dataset in this study. The Wisconsin dataset is a multivariate dataset that contains 569 instances of breast cancer, each with 30 input attributes, a single output attribute indicating malignant or benign tumor, and no missing values. The Wisconsin dataset was computed from a digitalized image of a fine needle aspirate of a breast mass [26], as shown in Figure 1. The total numbers of malignant and benign cancer cells indicated in the dataset are represented in Table 1, where 0 represents benign, and 1 denotes malignant. The dataset offers a well-validated database to explore breast cancer screening. Data collection from various sources is often raw and contains errors, outliers, or missing values. Hence, data preprocessing is necessary. Data preparation is usually expected to account for about 60% of the entire data mining process.

### 2.2. Data Pre-Processing

Data preprocessing is one of the most significant steps in ML and data mining [28]. It improves the quality of data and the performance of ML models. The dataset used for this study was preprocessed in a Jupyter notebook using the python programming language (python 3.8). Since there were neither missing nor null values, various techniques to remove and replace relevant values were not implemented on the Wisconsin dataset. Additionally, the dataset was relatively balanced; hence, oversampling or undersampling techniques were unnecessary. The dataset’s outliers were replaced with values from the 25th and 75th percentiles. This guaranteed that we maintained a sufficient quantity of usable datasets for our investigation. The appropriate handling of missing values and outliers helps eliminate redundancy, ambiguity, and noise, and promotes an accurate and reliable prediction model. We implemented the 10-fold cross-validation technique to evaluate the models to ensure that each fold of the dataset contained the same proportion of observations with a particular label. Finally, we employed principal component analysis (PCA) because the dataset had large dimensions. PCA is a dimensionality-reduction technique frequently used to reduce the dimensionality of large datasets. It does so by transforming a large group of variables into a smaller one that retains most of the information in the original dataset.

### 2.3. Supervised Learning Models

#### 2.3.1. Support Vector Machine (SVM)

Support Vector Machine (SVM) is a supervised learning technique for classifying data into two or more groups. SVM is primarily used for classification. However, it is seldomly used for regression. The optimal hyperplane is the best possible distance margin between two extreme points (support vector). It categorizes data by dividing it into two or more groups. In contrast, the distance margin is the distance between the support vectors and the hyperplane. An SVM model measures and maximizes the distance margin to achieve the best hyperplane. The optimal hyperplane is the one for which the margin is the greatest. If the hyperplane is not optimal (e.g., it has a low margin), misclassification is high. As a result, SVM seeks to establish a decision boundary that allows for as much separation between the two or more groups. A kernel function is employed to transform a non-linear dataset into one or two dimensions to identify the best hyperplane. SVM is unique because of its high dimensional input space, called the curse of dimensionality, sparse document vectors, and regularization parameter (λ).

#### 2.3.2. Random Forest

Random forest or random decision forest is an ensemble tree-based supervised learning method that operates independently by constructing multiple decision trees. Each tree in the random forest splits out a class prediction, and the class with the most votes becomes our model’s prediction. The random forest model can recreate body movement for object detection, remote sensing, and game console. It is used for both classification and regression scenarios. The fundamental principle behind random forest is a simple but powerful one. The random forest model builds multiple classifiers using randomly selected subsets of observation and random subsets of the predictor variables. The predictions from the tree ensemble are then tallied using a voting system for a classification tree. Its benefits include high accuracy, running efficiently on a large dataset, handling thousands of input variables without variable deletion, estimating variables significant in the classification, and offering an experimental method for detecting variable interactions.

#### 2.3.3. Logistic Regression

Logistic regression is a supervised learning model for performing binary classification. Logistic regression can predict if something is true/false or yes/no. Rather than fitting a line to the data as in linear regression, the line is fitted to an “S” shaped “logistic function”. Logistic regression is a useful machine learning technique due to its ability to generate probabilities and classify new samples using continuous and discrete variables. Its functioning is based on the notion of “maximum likelihood”, which seeks the optimal fit of a distribution to the data. Fitting a distribution (normal, exponential, or gamma) to data simplifies its manipulation.

#### 2.3.4. K-Nearest Neighbor (KNN)

KNN is a supervised machine learning model that is mainly used for classification. It classifies a data point according to the classification of its neighbors, maintains all available cases, and classifies new cases using a similarity metric (feature similarity). The “K” parameter denotes the number of nearest neighbors in the majority voting process. Choosing an appropriate value for K is referred to as parameter tuning, and it is critical for improved accuracy.

#### 2.3.5. Naive Bayes

The naive Bayes technique is a supervised learning model based on Bayes’ Theorem with the “naive” assumption of conditional independence between every pair of features given the class variable’s value. Naive Bayes is based on Bayes’ Theorem’s premise of conditional probability. It determines the conditional likelihood of an event occurring based on prior knowledge of possible event-related factors. Face recognition, weather forecasting, medical diagnosis, and news classification use naive Bayes. It is simple to build, requires less training data, works with both continuous and discrete data, is highly scalable in terms of predictors and data points, is insensitive to irrelevant features, and may be used for real-time prediction.

### 2.4. Performance Metrics

Performance metrics are part of every machine learning pipeline. Detailed information about the performance of the proposed techniques based on multiple criteria will be presented below.

#### 2.4.1. Confusion Matrix

**Figure 2:**
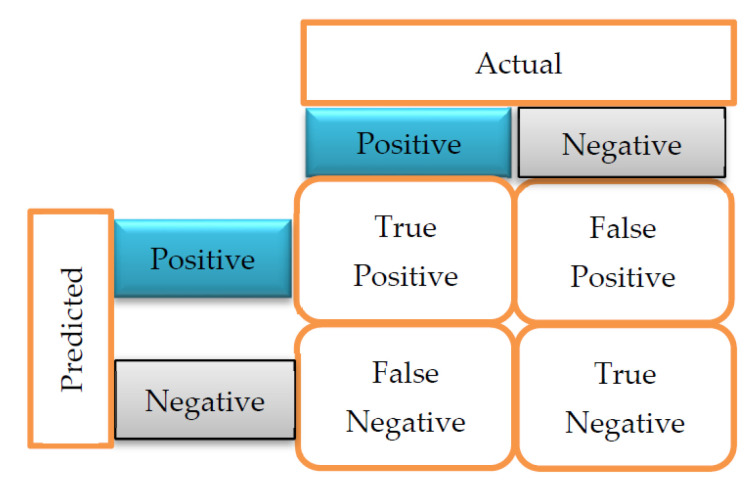
Confusion Matrix [17].

A confusion matrix is a table layout of the different outcomes of predictions and results of a classification problem. It helps visualize its outcome, as shown in Figure 2. The confusion matrix helps identify the correct predictions for different classes and errors.

True Positives (TP) indicate the number of times the actual positive values are equal to the predicted positive values. In contrast, True Negatives (TN) indicate the number of times the actual negative value equals the predicted negative value. False Positive (FP) is the number of times the model wrongly predicts negative values as positives. In contrast, False Negative (FN) is the number of times the model wrongly predicts positive values as negatives.

#### 2.4.2. Classification Accuracy

The most often used performance parameter for classification models is accuracy. It is used to determine the proportion of correctly categorized values, and it indicates how frequently the classification is correct. It can be calculated using:

(1) $$\mathrm{Accuracy}=\frac{\mathrm{TP}+\mathrm{TN}}{\mathrm{TP}+\mathrm{FP}+\mathrm{FN}+\mathrm{TN}}$$

#### 2.4.3. Classification Report

- Precision: is used to calculate the model’s ability to classify positive values correctly. It answers the question, “when the model predicts a positive value, how often is it right?” Precision is calculated using:
  (2) $$\mathrm{Precision}=\frac{\mathrm{TP}}{\mathrm{TP}+\mathrm{FP}}$$
- Recall or Sensitivity: Recall is the model’s ability to predict positive values. “How often does the model predict the correct positive values?” The equation for the recall is presented below:
  (3) $$\mathrm{Recall}=\frac{\mathrm{TP}}{\mathrm{TP}+\mathrm{FN}}$$
- Specificity: Specificity can be defined as the number of negatives returned by our machine learning (ML) model. It can be calculated by using:
  (4) $$\mathrm{Specificity}=\frac{\mathrm{TN}}{\mathrm{TN}+\mathrm{FP}}$$
- Support: Support may be defined as the number of samples of the true response in each class of target values.
- F1-Score/F-Measure: The F1-score is a weighted average of precision and recall (the harmonic mean of recall, and precision). The F1-scores can be calculated using:
  (5) $$F1-\mathrm{Score}\frac{2\times \left(\mathrm{Precision}\times \mathrm{Recall}\right)}{\mathrm{Precision}+\mathrm{Recall}}$$

### 2.5. Model Development

This phase entails the steps and processes undertaken after the pre-processing stage. Achieving the best tuning parameter for ML models can be a tedious undertaking. Thus, we employed the use of a SkLearn library function called GridSearchCV. This function helps to loop through pre-defined hyper-parameters and fit the model on the training set. Finally, the best parameters from the list are highlighted. Each model was assigned varying tuning parameters, and the parameters with the highest accuracy were selected. To obtain the optimal value of k for the kNN algorithm, we explored a range of 1 to 41 and achieved an optimal k value of 14. The logistic regression and naive Bayes model’s parameter were left at default, giving the best accuracy, while random forest recorded the highest accuracy with a n_estimator of 10. The SVM model produced the best result with a penalty parameter of the error term (c) as 10 and radial basis function (RBF) kernel. The training dataset was utilized to generate trained models for prediction throughout the model development phase of training. They were tested using the testing dataset to determine how accurate the models were in predicting the corresponding class labels in the testing dataset. Next, each model underwent 10-fold cross-validation (10-folds of training and testing with randomized data-split) to get an accurate model performance measurement. Finally, evaluation metrics were generated to compare the trained models.

### 2.6. Multi-Criteria Decision-Making Method (MCDM)

Multiple alternatives are a significant concern for decision-makers as they increase decision-making complexity. Hence, it is necessary to find a technique that reduces errors by incorporating influence decisions, decision-making processes, and criteria. Most of the time, these techniques are challenging to carry out since the criteria for making decisions are often in conflict, raising the ambiguity of the final result [29,30,31]. Subsequently, the emergence of multi-criteria decision-making methods has improved the reliability and credibility of the chosen solution [32,33]. Multi-criteria decision-making (MCDM), also referred to as multiple-criteria decision analysis (MCDA), is a research area that analyzes various available choices in a situation or research area which spans daily life, social sciences, engineering, medicine, and many other areas. It analyzes the criteria that make a parameter favorable or unfavorable for a particular application. MCDM aims to assist in the decision-making process, reduce the responsibilities attributed to decision-makers, and ensure a solution meets all criteria. In the health sector, these methods are much more complicated. They entail technical or economic considerations and the human factor, creating conflicts of interest and obstructing the final choice [31]. As a result, numerous instances of research utilizing MCDM have been conducted to optimize entire health systems [34,35,36,37,38,39,40].

Certain studies focus on a particular field, such as health technology evaluation [36], while others take a more humanitarian approach, examining research that seeks to ascertain patient preference [41]. Moreover, some studies go beyond, aiming to fully understand and study the MCDM’s application in health [42,43,44,45]. There are several MCDM methods which include the analytic hierarchy process (AHP), technique for order of preference by similarities to ideal solution (TOPSIS), elimination et choix traduisant la realité (ELECTRE), preference ranking organization method for enrichment of evaluations (PROMETHEE), visekriterijumska optimizcija i kaompromisno resenje (VIKOR), and data envelopment analysis (DEA). It is challenging to state which method is the best, as they all have advantages and disadvantages. However, recent studies have indicated the effective use of PROMETHEE in various medical applications. They include research on sterilization methods for medical devices [22] and the postexposure prophylaxis regimens for preventing pediatric HIV-1 infection [46]. Furthermore, the PROMETHEE technique possesses some advantages which make it unique when handling multiple criteria. These advantages include its ease of use, applicability to real-life problems, completeness in the ranking [47], and its ability to accommodate the use of both quantitative and qualitative data [48]. Hence, we adopt the PROMETHEE technique for our study.

We propose implementing this method with ML to compare alternatives based on selected criteria. This will be helpful to decision-makers in choosing an option with minimal compromise and maximum advantages. The criteria used in this study have both qualitative and quantitative values.

### 2.7. Fuzzy PROMETHEE

The preference ranking organization method for enrichment evaluations (PROMETHEE) is an MCDM tool that enables users to examine and rank alternatives according to their criteria. The PROMETHEE method was developed by Brans and Vince in 1985 [49] to compare available alternatives based on the selected criteria [50]. It is favored over other MCDM techniques, such as the analytic hierarchy process and the methodology for ordering performance according to resemblance to the ideal solution [51]. It enables the user to exert complete control on the preference values of the criteria [52]. PROMETHEE is one of the most popular decision-making tools utilized in various fields [52,53,54]. It requires only a few pieces of information from the decision-maker: the weights assigned to the specified criteria and the preference function to assess the superiority of the alternative on each criterion [52].

Fuzzy logic can be characterized in its simplest form as a decision mechanism design. It enables decision-makers to determine vague conditions and, when necessary, examine systems using linguistic data [55]. Following Zadeh’s proposal in 1965 and definition of fuzzy set theory, researchers in various fields have studied hybrid models of classical models, referred to as fuzzy-based models. Because many objects and cases in the actual world lack crisp distinctions, it has been determined that characterizing and modeling problems using fuzzy sets can result in a more responsive model to real-world difficulties. Fuzzy-based MCDM is more suitable for several cases where numerical data are unavailable. Moreover, it allows decision-makers to analyze alternatives in linguistic data [56].

To evaluate the alternatives, a variety of criteria are proposed. They include accuracy, recall, precision, F1-score, receiver operating characteristic curve-area under the curve (ROC-AUC), log loss, number of samples needed, the impact of feature scaling, hyper-parameter tuning, and tolerance to irrelevant attributes. Accuracy, recall, precision, F1-score, receiver operating characteristic curve–area under the curve (ROC-AUC), and log loss are the performance indicators most frequently employed in ML [57]. Previous studies have used these indicators to evaluate the best ML models for use in breast cancer [58,59,60]. These criteria are selected because they are important factors when choosing an appropriate ML model for use. Ideally, more training datasets result in lower test errors (model variance decreases, indicating less overfitting). Theoretically, having more data does not always result in a more accurate model, as high-bias models do not benefit from additional training data. While some ML models work well with large datasets, others are only efficient when the available data are small [61]. Moreover, ML models vary in their tolerance to irrelevant attributes. Some models perform well in the presence of noise and other irrelevant information, while others are greatly affected by them. Criteria are generally used as a bottom line for comparing alternatives [62]. To apply fuzzy PROMETHEE, each criterion is simplified using a linguistic scale of importance. The weight was selected based on the importance of each criterion with respect to the expert’s opinion.

Choosing the criteria upon which alternatives will be evaluated in the decision-making process is vital. Because not all criteria are equally significant, assigning weights to establish a relative priority is necessary. This indicates that the most important criteria are given a higher weight, while the least critical criteria are given a lower weight. Weighting is typically used to prioritize important criteria and highlight their relative importance by assigning a weight to each. However, scientific research frequently requires expert opinion to determine which criteria should be given more or less priority. Even though the study’s co-authors are experts in medicine, laboratory medicine, engineering, and mathematics, we consulted other experts in surgical oncology, breast surgery, radiology and radiography, pathology, and radiation oncology. This ensured that we did not leave any stone unturned while weighting all criteria.

Assigning weight to several criteria may differ from one decision-maker to another, which is one of the uniquely important elements of the MCDM technique. When deploying a model for breast cancer detection, it is crucial to know if the model predicts values correctly or not, as this will hugely impact treatment. A decision-maker would not want to commence treatment without a disease, thereby endangering the patient’s life. Moreover, a decision-maker will want to know the wrong predictions made by the model and how many there are. Accuracy, recall, precision, and F1-score are a few of the most commonly used evaluation metrics to depict this scenario. They are the criteria that most directly portray and impact the model’s performance, indicating correctly and incorrectly classified values. As a result, they were assigned a very high weight as shown in Table 2. The number of training samples needed is also important because there is a chance of noise that may impact the algorithm’s performance and sometimes cause the crash of the machine, among other things. Moreover, it has been demonstrated that a larger dataset directly influences estimation variance and results in a model’s better predictive performance [63]. Thus, the number of training samples needed was assigned a high weight. Feature scaling was used to ensure that all of the data’s independent features were on about the same scale, making it easier for most ML algorithms to handle the data. An ML algorithm weighs greater values higher and smaller values lower regardless of the value’s unit if feature scaling is not completed. In ML, hyperparameters are parameters whose values govern the learning process and thus control the model’s overall behavior. Hyperparameter optimization is all about finding a combination of hyperparameters that will deliver the best possible results. Therefore, feature scaling and hyperparameter tuning significantly impact model performance and were also assigned a high weight. Irrelevant attributes mislead the training process by increasing training time and error variance resulting in bias. This ultimately affects the model’s performance. Tolerance to irrelevant attributes is important, but not as much as the previously stated criteria, so it was assigned a medium weight. All the criteria were distributed on a numerical scale of 0 to 1.

The gaussian preference function was applied to prevent minor deviations in the input values of the parameters. Afterward, the Yager index was used to defuzzify the triangular fuzzy set to enable an appropriate weight determination of each criterion [64]. The Yager index is a preferred method for defuzzification because it allows the decision-maker to compare fuzzy values rationally. The preference function adopted for this study is the Gaussian function for each criterion. This is because it provides a continuous probability distribution that is symmetrical around its mean and fits many natural phenomena.

## 3. Results and Discussion

The implementation tools and platforms of the ML models include python, pandas, NumPy, matplotlib, and Jupiter notebook. Table 3 shows the decision matrix of alternatives to be imputed into the PROMETHEE system for model evaluation. The accuracy, recall, precision, F1-score, ROC AUC, and log loss values were obtained from the model development, training, and prediction phase. The records for the impact of missing and imbalanced datasets, irrelevant attributes, hyper-parameter tuning, and feature scaling were obtained from the previous research literature. SVM outperformed other models with the highest accuracy of 99.0% and lowest log loss of −0.828, whereas naive Bayes, random forest, and logistic obtained the lowest accuracy of 97.5% and highest log loss of −0.815. Log loss is one of the most important classification metrics based on probability, and lower log-loss values mean better prediction. Even with the lowest accuracy of 97.5%, the results obtained were satisfactory. This makes the model entirely appropriate and satisfactory to implement in the early detection of breast cancer. The recall (sensitivity) for all the models was also satisfactory. The SVM performed best with a recall of 99.0%. The KNN model had a recall of 98.0%, while random forest, logistic regression, and naive Bayes had a lower recall of 97.0%, respectively.

When compared with previous studies employing the Wisconsin breast cancer (diagnostic) dataset, it is clear that our proposed method outperforms them. The result obtained from the study conducted by Ak [65] indicated that logistic regression outperformed all other models, including KNN, SVM, naive Bayes, and random forest, with the highest classification accuracy of 98.1%. This is lower than the 99.0% highest classification accuracy recorded by our most preferred model (SVM). Moreover, Kaklamanis and Filippakis [66] indicated that KNN, with the highest accuracy of 96.5% and kappa of 92.4%, performed better than other models such as CART, naive Bayes, and SVM radial basis function (RBF) kernel. Naive Bayes recorded the lowest accuracy, in addition to kappa, of 88.3% and 74.4%, making it the least preferred alternative. In contrast, the accuracy of KNN in our study was about 1.5% higher. Moreover, in contrast to the two performance metrics used in that research, our study implemented more performance metrics.

The accuracy and recall of each model were then analyzed with other criteria, including the number of training samples needed and tolerance to irrelevant attributes to fully evaluate which model was the most preferred in this circumstance using fuzzy PROMETHEE. A model with low accuracy and high tolerance to irrelevant attributes is unsuitable, so there should be balance when selecting an appropriate model. The reliance on accuracy, recall, and specificity for choosing the best model can be faulty depending on the scenario.

With a net flow of 0.3954, SVM was determined as the most favorable and preferred model for the early detection of breast cancer using the Wisconsin breast cancer dataset. KNN, logistic regression, and the random forest classifier came second, third, and fourth, with net flows of 0.0845, −0.0841, and −0.1401, respectively. Naive Bayes with a net flow of −0.2557 was the least preferred alternative, as shown in Table 4. However, the results may differ if a different weight is assigned to each criterion.

**Figure 3:**
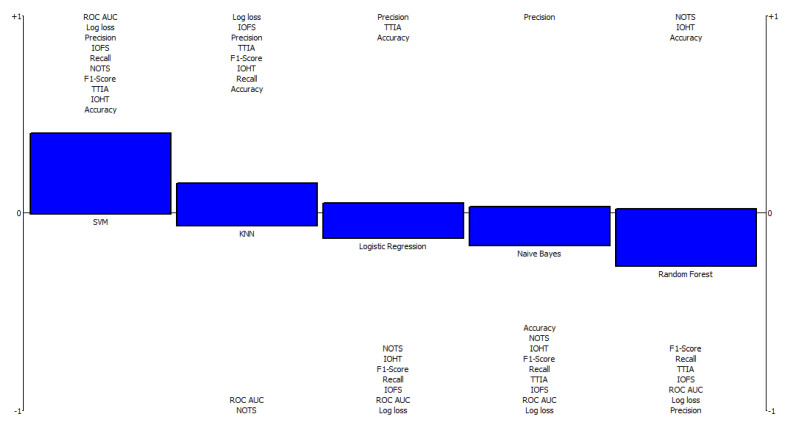
Rainbow ranking of machine learning algorithms. NOTS: Number of training samples; IOFS: impact of feature scaling; IOHT: impact of hyper-parameter tuning; TTIA: tolerance to irrelevant attributes; ROC-AUC: receiver operating characteristic curve–the area under the curve.

Figure 3 shows a comprehensive rainbow ranking of the model, indicating its strengths and weaknesses and the final ranking of alternatives. This graph showcases a representation of each model from the most preferred to the least preferred. The values above the 0 threshold represent the alternatives’ strengths, while the parameters below the 0 threshold represent their weaknesses. The rainbow diagram illustrates the net flow values where alternatives are displayed from left to right based on their ranks. The alternatives are depicted by a vertical bar made up of criteria. Every section of this bar represents how a single criterion contributes to determining an alternative’s total net flow value. The height of the vertical bar shows the difference between the positive and negative preference flow, multiplied by the corresponding weight of the provided criterion. The indicators at the top of the vertical bar have the highest positive values, while the indicators at the bottom of the vertical bar have the highest negative values. As a result, the PROMETHEE rainbow diagram provides a comprehensive overview of all alternatives and criteria, including the relative importance of each.

Furthermore, we tested our approach on the BIRADS dataset [67]. The BIRADS dataset contains a mammographic mass of 961 instances, 6 attributes, 516 benign, and 445 malignant breast cancer patients. Data pre-processing was carried out to remove missing and null values, and the outliers present were replaced with values in the 25th and 75th percentile. We obtained results similar to those achieved using the Wisconsin dataset, and the decision matrix of alternatives was designed as shown in Table 5. The SVM model had a significantly higher values of 97.0%, 96.5%, 97.5%, and 98.5% for accuracy, recall, precision, and F1-score. The naive Bayes model recorded the lowest value of 94.0% for both accuracy and precision but a substantially higher value for tolerance to irrelevant attributes. Moreover, the negative log loss of −0.8110 obtained by the SVM model further proves its outranking performance compared with other models. After applying the same weight and preference function to the criteria, we obtained a complete ranking of alternatives, as shown in Table 6. The rankings indicated similarity in the results obtained when the Wisconsin dataset was used. The SVM ranked top among other alternatives with a net outranking flow of 0.1022. The KNN model ranked second with a net outranking flow of 0.0316, while the logistic regression, random forest, and naive Bayes classifier ranked third, fourth, and fifth with a net outranking flow of −0.0032, −0.0541, and −0.0766, respectively. The SVM outranked other models when the two datasets were used. This indicates that our approach is applicable and efficient in evaluating model performance. The results may differ if the weight assigned to different criteria is altered. The result obtained indicates the applicability and use of the MCDM approach in model selection.

**Figure 4:**
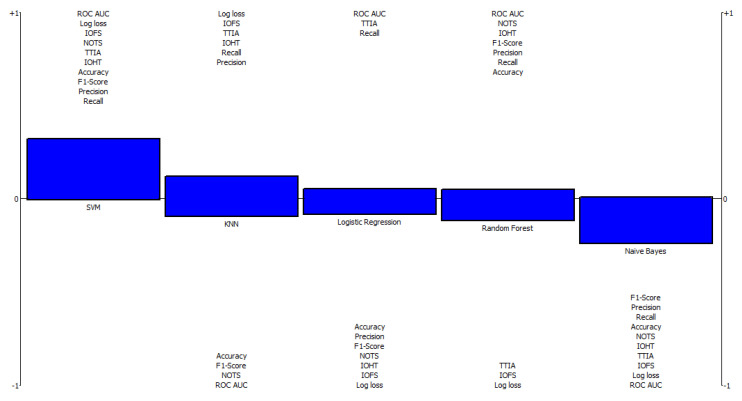
Rainbow ranking of ML algorithms using the BIRADS dataset. NOTS: number of training samples; IOFS: impact of feature scaling; IOHT: impact of hyper-parameter tuning; TTIA: tolerance to irrelevant attributes; ROC-AUC: receiver operating characteristic curve–the area under the curve.

As shown in Figure 4, the rainbow ranking indicates the highest positive and negative values for all alternatives. The SVM model has the highest positive values for the impact of feature scaling, the number of training samples needed, tolerance to irrelevant attributes, the impact of hyper-parameter tuning, accuracy, F1-score, precision, and recall. Subsequently, the naive Bayes model recorded the highest negative values for F1-score, precision, recall, accuracy, the number of the training samples, the impact of hyper-parameter tuning, tolerance to irrelevant attributes, and the impact of feature scaling.

## 4. Conclusions

This study proposes a novel approach to determine the most appropriate ML model for breast cancer screening. This novel approach takes the evaluation of ML models to the next step by incorporating more factors than simply the often-used performance metrics, and therefore creates a new path to model evaluation. Recently, decision-makers have sought a more robust method for incorporating metrics other than accuracy, precision, recall, F1-score, ROC-AUC, and log loss into their model selection process. The findings in this study provide that. This study considers other important criteria, such as the number of samples needed, the impact of feature scaling, hyper-parameter tuning, and tolerance to irrelevant attributes. The results of this study show that these criteria matter.

## Figures and Tables

**Table 1:** Class Distribution.

	Label	Count	Designation
1	Malignant (M)	212	1
2	Benign (B)	357	0

**Table 2:** The linguistic scale of importance.

Linguistic Scale	Triangular Fuzzy Scale	Criteria
Very High (VH)	(0.75, 1, 1)	Accuracy, recall, precision F1-score, ROC-AUC, log loss
High (H)	(0.50, 0.75, 1)	Number of training samples needed, the impact of feature scaling, the impact of hyper-parameter tuning
Medium (M)	(0.25, 0.50, 0.75)	Tolerance to irrelevant attributes
Low (L)	(0, 0.25, 0.50)	-
Very Low (VL)	(0, 0, 0.25)	-

**Table 3:** Decision matrix of alternatives.

Criteria	Accuracy	Recall	Precision	F1-Score	ROC AUC	Log Loss	Number of Training Samples Needed	Impact of Feature Scaling	Impact of Hyperparameter Tuning	Tolerance to İrrelevant Attributes
SVM	99.0%	99.0%	99.5%	99.0%	99.5%	−0.828	0.92	0.92	YES	0.92
Random Forest	97.5%	97.0%	98.0%	97.0%	99.0%	−0.815	0.75	0.08	YES	0.08
Logistic Regression	97.5%	97.0%	98.0%	97.0%	99.0%	−0.815	0.50	0.25	NO	0.50
KNN	98.0%	98.0%	98.5%	98.0%	99.0%	−0.819	0.08	0.92	YES	0.50
Naive Bayes	97.5%	97.0%	98.0%	97.0%	99.0%	−0.815	0.50	0.08	NO	0.75

**Table 4:** The complete ranking of alternatives using the Wisconsin dataset.

Ranking	Alternatives	Positive Outranking Flow	Negative Outranking Flow	Net Flow
1	SVM	0.3954	0.0000	0.3954
2	KNN	0.1807	0.0962	0.0845
3	Logistic Regression	0.0516	0.1357	−0.0841
4	Naive Bayes	0.1401	0.1718	−0.1401
5	Random Forest	0.013644	0.2693	−0.2557

**Table 5:** Decision matrix of alternatives for the BIRADS dataset.

Criteria	Accuracy	Recall	Precision	F1-Score	ROC AUC	Log Loss	Number of Training Samples Needed	Impact of Feature Scaling	Impact of Hyperparameter Tuning	Tolerance to İrrelevant Attributes
SVM	97.0%	95.5%	97.5%	98.5%	99.5%	−0.8110	0.92	0.92	YES	0.92
Random Forest	96.0%	96.0%	98.0%	98.0%	99.0%	−0.8026	0.75	0.08	YES	0.08
Logistic Regression	95.5%	95.5%	97.0%	96.5%	99.0%	−0.7984	0.50	0.25	NO	0.50
KNN	95.5%	96.0%	97.5%	96.0%	98.5%	−0.7990	0.08	0.92	YES	0.50
Naive Bayes	94.0%	94.0%	96.0%	96.0%	98.0%	−0.7860	0.50	0.08	NO	0.75

**Table 6:** The complete ranking of alternatives using the BIRADS dataset.

Ranking	Alternatives	Positive Outranking Flow	Negative Outranking Flow	Net Flow
1	SVM	0.315	0.0000	0.3152
2	KNN	0.1734	0.1493	0.0241
3	Logistic Regression	0.08336	0.1157	−0.0321
4	Random Forest	0.0729	0.1438	−0.0709
5	Naive Bayes	0.0044	0.2408	−0.2363

## Data Availability

The dataset used were anonymous data obtained from https://archive.ics.uci.edu/ml/datasets/breast+cancer+wisconsin+(diagnostic) (Last Accessed on 15 January 2022) and http://archive.ics.uci.edu/ml/datasets/Mammographic+Mass (Last Accessed on 15 January 2022).
